# Classifying simulated gait impairments using privacy-preserving explainable artificial intelligence and mobile phone videos

**DOI:** 10.1371/journal.pdig.0001004

**Published:** 2025-09-16

**Authors:** Lauhitya Reddy, Ketan Anand, Shoibolina Kaushik, Corey Rodrigo, J. Lucas McKay, Trisha M. Kesar, Hyeokhyen Kwon

**Affiliations:** 1 Department of Biomedical Engineering, Emory University and Georgia Institute of Technology, Atlanta, Georgia, United States of America; 2 Department of Electrical and Computer Engineering, Georgia Institute of Technology, Atlanta, Georgia, United States of America; 3 Department of Computer Science, Emory University, Atlanta, Georgia, United States of America; 4 Department of Rehabilitation Medicine, Emory University, Atlanta, Georgia, United States of America; 5 Department of Neurology, Emory University, Atlanta, Georgia, United States of America; 6 Department of Biomedical Informatics, Emory University, Atlanta, Georgia, United States of America; The University of Hong Kong, HONG KONG

## Abstract

Accurate diagnosis of gait impairments is often hindered by subjective or costly assessment methods, with current solutions relying on either expensive multi-camera equipment or subjective clinical observation. There is a critical need for accessible, objective tools that can aid in gait assessment while preserving patient privacy. In this work, we present a mobile phone-based, privacy-preserving artificial intelligence (AI) system for classifying gait impairments that leverages a novel dataset of 743 videos capturing seven distinct gait types. The dataset consists of frontal and sagittal views of clinicians simulating normal gait and six types of pathological gait (circumduction, Trendelenburg, antalgic, crouch, Parkinsonian, and vaulting), recorded using standard mobile phone cameras. Our system achieved 86.5% accuracy using combined frontal and sagittal views, with sagittal views generally outperforming frontal views except for specific gait types like circumduction. Model feature importance analysis revealed that frequency-domain features and entropy measures were critical for classification performance. Specifically, lower limb keypoints proved most important for classification, aligning with clinical understanding of gait assessment. These findings demonstrate that mobile phone-based systems can effectively classify diverse gait types while preserving privacy through on-device processing. The high accuracy achieved using simulated gait data suggests their potential for rapid prototyping of gait analysis systems, though clinical validation with patient data remains necessary. This work represents a significant step toward accessible, objective gait assessment tools for clinical, community, and tele-rehabilitation settings.

## Author summary

Our research aims to make gait analysis more accessible and private by using pose estimates recorded on a standard mobile phone. Gait, or the way people walk, is crucial for diagnosing movement impairments caused by conditions like stroke or Parkinson’s disease. Currently, accurate gait assessment often requires expensive equipment or subjective judgments by specialists. Our approach instead uses a mobile phone, allowing us to gather video data that can be processed directly on the device, protecting individuals’ privacy while achieving reliable results. In our study, we video recorded trained clinicians simulating different types of gait impairments to create a diverse dataset, which we used to train the AI system to recognize and classify these gait types. Our system can correctly identify various simulated gait types. Our findings demonstrate that mobile phones can serve as effective, low-cost tools for clinical gait analysis. With further validation, this technology could become a practical solution for monitoring and diagnosing movement disorders in everyday environments, supporting broader access to health monitoring and rehabilitation.

## Introduction

Walking is essential for functional mobility and activities of daily living [1]. Gait impairments differ significantly in class and severity, depending on the individual’s specific neuropathology, such as stroke, Parkinson’s disease, spinal cord injury, or traumatic brain injury [1]. This variability in classes and causes of gait impairments poses significant challenges in precise diagnosis when using observational gait analysis in clinical settings [2–4], a method reliant on visual assessment and interpretation of gait. Although observational analysis is common in clinical settings, it has limited accuracy, test-retest, and inter-rater reliability [5,6]. For objective, accurate, and sensitive gait assessments, marker-based 3D gait analysis is the gold standard method used in motion analysis laboratories and academic medical centers [7,8]. However, the adoption of marker-based 3D motion capture is limited by high costs, the need for specialized expertise, and time constraints [9,10].

Recently, with advances in computer vision techniques, numerous studies have explored markerless gait analysis methods [11,12]. These novel methods eliminate the need for markers and expensive equipment by utilizing pose estimation algorithms [13,14] that extract the locations of anatomical key points from only videos recorded using low-cost video cameras. The markerless approach reduces both time and setup complexity for gait analysis. Several recent studies have demonstrated the clinical value of deriving gait kinematics from pose estimation for quantifying gait types and classifying pathological conditions, thus enhancing diagnostic and prognostic value [11,12,15,16]. Pose estimation, therefore, may overcome the limitations of both laboratory-based high-tech, high-cost 3D gait analysis and clinic-based, low-tech observational gait analysis, enhancing the accessibility, scalability, and reliability of gait assessments.

Although an area of active research, recent studies have primarily employed markerless methods for a limited range of pathological gaits–including stroke, amputation, Parkinson’s Disease, and Cerebral Palsy [15–18]–and no studies have investigated the use of datasets containing more than three gait classes. To understand the applicability of markerless gait analysis, there is a need for benchmark datasets that include not only normal and one type of impaired gait, but instead large datasets comprising a wide variety of pathological gait classes. However, these video datasets are challenging to collect, annotate, and analyze. Such benchmark datasets of different types of pathological gait, if available, will help provide a fair comparison between various pose estimation algorithms, and gait analysis methodologies for the research community. Furthermore, most existing studies that employ pose estimation for analysis of human gait videos assume the availability of a secure computer network with a graphical processing unit (GPU)-enabled cloud servers for processing gait video data [17,18]. This poses a challenge with respect to preserving participant privacy, as video data can capture sensitive information, such as bystanders, nudity, facial features, and other potential identifiers (like tattoos) when used in the real world. A recent IBM report stated that security and privacy data breaches related to cloud computing could cost $4.45 million per incident [19]. Finally, most studies using pose estimation algorithms alongside AI systems utilize a black box approach, lacking interpretability and limiting their use for clinical decision-making, by preventing clinicians from understanding potential biases or limitations in the AI models and how they arrive at their conclusions.

To address these gaps in markerless approaches, the objective of our current work was to demonstrate that pose estimation models running on a mobile-phone-based system [13,20] can classify various types of gait pathologies simulated by clinical specialists. We hypothesized that pose landmarks extracted independently from two mobile-phone videos—one frontal and one sagittal—can be fused to improve gait-type classification accuracy, without any added complication or time for synchronizing a dual camera setup. This work provides a significant step toward the long-term vision of implementing scalable, accessible, robust, privacy-preserving, and interpretable gait assessments in clinical, home, and community environments for diagnosing gait dysfunction and tracking gait recovery.

## Materials and methods

### Ethics statement

All study procedures were approved by the Human Subjects Institutional Review Board of Emory University (IRB Approval Number: 00003848). Participants provided written informed consent before participating in the gait video data collection.

### Study design and video dataset

**Fig 1:**
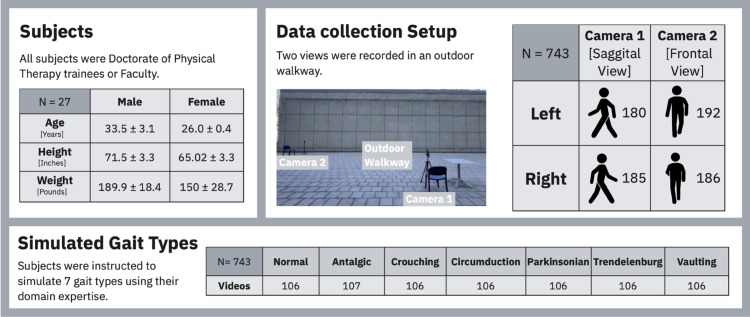
The demographics of our trained able bodied clinicians, setup of our video data collection, and number of simulated gait types.

We collected video data from 27 able-bodied participants (4 males, mean age 26±0.4; 23 females, mean age 33.5±3.1), all of whom were Doctor of Physical Therapy (DPT) students or faculty (See Fig 1 for Participant demographics). Participants walked an outdoor overground ten meter straight path with a camera placed perpendicularly four meter from the walking line. This walking distance, commonly used in clinical gait assessments, captures five to ten consecutive gait cycles. Each video yielded, on average, seven gait cycles. The total number of cycles observed across all videos varied by gait type per video (ANT: 7.6; CIR: 5.8; CRO: 7.2; NOR: 4.6; PAR: 10.4; TRE: 6.4; VAU: 5.4), reflecting individual differences in stride length and impairment severity. Participants completed one trial for each of the seven simulated gait classes: normal (NOR), circumduction (CIR), Trendelenburg (TRE), antalgic (ANT), crouch (CRO), Parkinsonian (PAR), and vaulting (VAU). These seven gait patterns were selected based on clinically important and commonly observed gait impairments in neurological populations—as defined by physical-therapy experts—to ensure representation of diverse types of gait impairments in a non-lab setting [21–25].

Participant inclusion criteria included the ability to follow instructions, no medical or musculoskeletal conditions interfering with walking, and the ability to accurately (as judged by the experimenters) replicate the simulated gait impairments. The number of videos per gait class is summarized in Fig 1: Simulated Gait Types. Before each trial, participants received verbal instructions on how to simulate each gait impairment, with asymmetrical gaits (e.g., CIR, ANT) performed as if the right leg was affected. For each trial, we recorded separate videos for walks in two directions (left and right) and from two camera angles (frontal and sagittal views). From the frontal view camera’s perspective, when walking towards the *Right*, participants moved away from the camera (camera facing their back); when walking to the *Left*, participants moved towards the camera (camera facing their front). A total of 743 gait videos were recorded, evenly split between frontal and sagittal views. The number of videos in each view and direction are summarized in Fig 1: Data collection Setup.

### Privacy-preserving video-based gait analysis

#### Overall pipeline.

**Fig 2:**
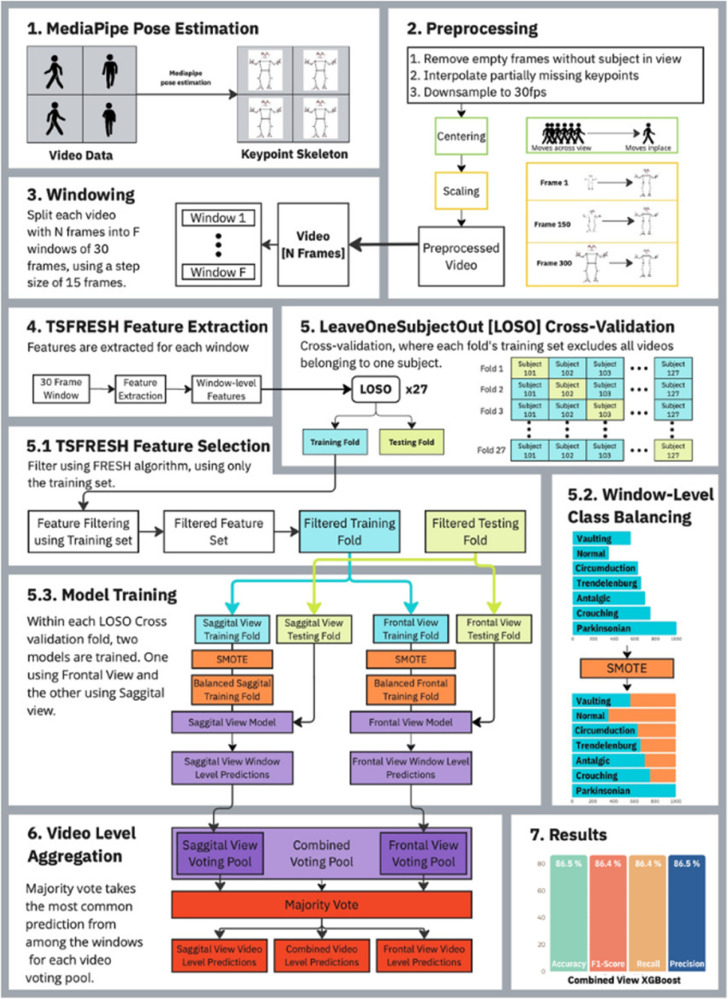
Overall video-based gait analysis pipeline and evaluation approach.

Our analysis pipeline (Fig 2) followed a standard human activity recognition framework [26]. We used Mediapipe [20] for on-device pose estimation [13], preprocessed the poses, segmented them into overlapping 1-second analysis frames, extracted statistical, frequency time series features, and trained Support Vector Machine (SVM) [27], Random Forest (RF) [28], and Extreme Gradient Boosting (XGBoost) [29] classifiers to make predictions for each analysis frame. Video-level gait class predictions were made by aggregating frame-level predictions using majority voting.

#### Preprocessing.

Pose estimation was conducted using MediaPipe [13,20], extracting the position of 33 keypoints across time from each video(Fig 2; 1. MediaPipe Pose Estimation). Each keypoint within a frame was represented by three channels: its x and y pixel coordinates, along with the estimated depth or z-axis distance from the camera. The pose estimator was run on-device, a setup allowing for only the detected poses to be uploaded to the server, ensuring that identifiable or sensitive data, such as the face or any nudity, would be safeguarded from potential data breaches. Frames where the participant was not visible or where no pose was detected were excluded from the analysis. For poses with partially missing keypoints due to occlusions (e.g., the arm facing away from the camera being obstructed by the torso), missing keypoints were linearly interpolated over time. All the pose sequences were downsampled to 30 frames per second (fps), as the majority of low-spec mobile phone cameras support 30 fps, improving the scalability of the developed pipeline.

Next, all poses were projected to a hip-center coordinate. This process removed potential biases in the model, arising from variations in the detected pose’s relative position to the camera. To preserve the natural sway of the hips during movement, we centered the keypoints using the median of the hip location from a 2-second (60 frames) window centered at the corresponding frame. For the frontal view, we re-scaled poses to the same height, removing perspective biases caused by changes in size due to the individual’s distance from the camera. Participants further away from the camera appeared smaller, which necessitated this re-scaling. For the sagittal view, we skipped this re-scaling process, as the participant’s perspective size and distance from the camera remained nearly constant throughout the video sequence. The centering and re-scaling processes were illustrated in Fig 2 (2. Preprocessing). Finally, the preprocessed pose sequences were segmented into 1-second windows (analysis frames) using a sliding window with 30 frames (1 second) and 50% overlap Fig 2 (3. Windowing). We chose a 1-second window following previous work in human activity recognition for gait classification [30].

#### Gait impairment video classification.

#### Extraction and selection of features.

For each 1-second analysis, we extracted time series features from each keypoint to characterize the gait window. Specifically, we used Time Series FeatuRe Extraction based on Scalable Hypothesis tests (TSFRESH) [31] Python package, a widely used time series feature extraction pipeline, to generate 783 features per channel [31]. In total, we extracted 77,517 features from 99 keypoint time series. These features were then reduced using the Fresh (FeatuRe Extraction and Scalable Hypothesis testing) algorithm [32], which eliminates time series features that are statistically insignificant to classification tasks. The Fresh algorithm utilizes the Benjamini-Yekutieli (BY) method [33], which identifies high dependencies or autocorrelation in time series features. Feature selection reduced the set of feature extraction methods used in the sagittal view to 31 and in the frontal view to 37, as included in Table 1.

**Table 1:** Comparison of selected feature types across sagittal and frontal views. **Legend:** ${\;}^{†\text{S}}$    Not relevant in Sagittal View as per TSFresh   ${\;}^{†\text{F}}$    Not relevant in Frontal View as per TSFresh

Feature Type	Definition
abs energy	Returns the absolute energy of the time series, which is the sum over the squared values.
absolute maximum	Calculates the highest absolute value of the time series *x*.
absolute sum of changes	Returns the sum over the absolute value of consecutive changes in the series *x*.
agg autocorrelation	Descriptive statistics on the autocorrelation of the time series.
agg linear trend	Calculates a linear least-squares regression for values of the time series that were aggregated over chunks versus the sequence from 0 up to the number of chunks minus one.
approximate entropy	Quantifies the amount of regularity and unpredictability of fluctuations over the channel.
autocorrelation	Calculates the autocorrelation of the specified lag, according to the formula.
benford correlation	Returns the correlation from first digit distribution; useful for anomaly detection applications.
binned entropy${\;}^{†\text{S}}$	Bins the values of *x* into a maximum number of equidistant bins.
c3	Uses c3 statistics to measure non-linearity in the time series.
change quantiles	Fixes a corridor given by the quantiles $q_{l}\text{and}q_{h}\text{of}\mathrm{the}\text{distribution}\mathrm{of}x$.
cid ce	Estimates time series complexity (complex time series have more peaks, valleys, etc.).
count above${\;}^{†\text{S}}$	Returns the percentage of values in xthatarehigherthanathresholdt.
count below${\;}^{†\text{S}}$	Returns the percentage of values in xthatarelowerthanathresholdt.
cwt coefficients	Calculates a Continuous Wavelet Transform for the Ricker wavelet (Mexican hat wavelet).
fft aggregated	Returns the spectral centroid, variance, skew, and kurtosis of the absolute Fourier transform spectrum.
fft coefficient	Calculates the Fourier coefficients of the one-dimensional discrete Fourier Transform for real input by FFT algorithm.
fourier entropy${\;}^{†\text{S}}$	Calculates the binned entropy of the power spectral density of the time series (using the Welch method).
linear trend	Calculates a linear least-squares regression for the time series versus the sequence from 0 to the length of the time series.
max langevin fixed point	Largest fixed point of dynamics estimated from polynomial h(x).
maximum	Calculates the highest value of the time series *x*.
mean	Returns the mean of *x*.
mean abs change	Average over first differences in the time series.
mean n absolute max	Calculates the arithmetic mean of the *n* absolute maximum values of the time series.
median	Returns the median of *x*.
minimum	Calculates the lowest value of the time series *x*.
number crossing m	Calculates the number of crossings of xonathresholdm.
number peaks${\;}^{†\text{S}}$	Calculates the number of peaks of at least support ninthetimeseriesx.
permutation entropy	Calculates the permutation entropy for time series complexity.
quantile	Calculates the qquantileofx.
range count${\;}^{†\text{S}}$	Counts observed values within the interval [min,max).
root mean square	Returns the root mean square (RMS) of the time series.
sample entropy	Calculates and returns the sample entropy of *x*.
standard deviation	Returns the standard deviation of *x*.
sum values	Calculates the sum over the time series values.
variance	Returns the variance of *x*.
variation coefficient	Returns the variation coefficient (standard error / mean) for *x*.

#### Data augmentation.

The different walking speeds observed across simulated gait classes and participants led to an imbalance in the number of segmented windows per gait class, as displayed in Fig 2 (5.2 Window-level Class Balancing). Class imbalance could potentially result in suboptimal performance of machine learning models [34]. To address this, we applied the Synthetic Minority Over-sampling Technique (SMOTE) [35], which increased the sample sizes of the minority gait classes (VAU, NOR, CIR, TRE, ANT, and CRO) to match those of the PAR gait class. SMOTE oversampled existing minority class examples by generating new synthetic samples by interpolating across their features, helping to mitigate class imbalance and improve model performance.

#### Video-level classification.

The augmented window-level features were input into several machine learning models previously used in gait research [36–38], which were SVM [27], RF [28], and XGBoost [29], to classify each window to a specific gait class. For single view video-level classification, we aggregated the labels inferred from each window in the video using a majority voting scheme. For multi-view video-level classification, majority voting was applied across all window-level labels from both frontal and sagittal views of the participant simulating the gait. The video-level aggregation was illustrated in Fig 2 (6. Video-level Aggregation).

#### Experiment and evaluation.

#### Cross validation and evaluation metrics.

To validate the proposed gait classification pipeline, we used a user-independent nested cross-validation approach following the standard methods in machine learning research [39,40]. This was to validate the generalizability of the trained model when tested on unseen participants, separate from the training set. First, we split the participant data using a Leave-One-Subject-Out (LOSO) cross-validation scheme, where each fold used data from one participant as the test set and the remaining participants’ as the training set (Outer Loop). Within each fold, the training set was further divided using a user-independent 5-fold cross-validation scheme, where 20% of the participants were used as the validation set and the remainder as the training set (Inner Loop). The inner loop was used to tune hyperparameters of each model (SVM, RF, and XGBoost), listed in Table 2, using an open-source package named Optuna [41]. The outer loop’s test set was used to derive the final model evaluation. The Fresh feature selection and SMOTE techniques were applied exclusively to the training set to prevent information leakage from the validation set (in the inner loop) and the test set (in the outer loop) during nested cross-validation. Model performance was evaluated using accuracy, F1 score, precision, and recall. To evaluate the statistical significance of the model performances, we applied ten trials of nested cross-validation and reported the average test scores across all splits, accompanied by 95% confidence intervals based on Z-type or normal approximation type confidence intervals [42].

**Table 2:** Hyperparameters tuned for SVM, RF, and XGBoost models.

Model	Hyperparameter	Description
SVM	C	Regularization parameter controlling trade-off between error and margin
	gamma	Defines how far the influence of a single training example reaches
	kernel	Specifies the kernel type to be used in the algorithm (linear or rbf, in this case)
RF	n_estimators	Number of trees in the forest
	max_depth	Maximum depth of the tree
	min_samples_split	Minimum number of samples required to split an internal node
	min_samples_leaf	Minimum number of samples required to be at a leaf node
	max_features	Number of features to consider when looking for the best split
XGBoost	max_depth	Maximum depth of a tree in the model
	learning_rate	Step size shrinkage to prevent overfitting
	n_estimators	Number of boosting rounds (trees)
	min_child_weight	Minimum sum of instance weight needed in a child node
	subsample	Fraction of samples used for building each tree
	colsample_bytree	Fraction of features used when building each tree

#### Multi-class and per-gait classification tasks.

We first studied the overall multi-class classification performance for all seven gait classes. Then, we studied the per-gait classification performance when considering one gait class as positive samples while considering all other six gait samples as negative samples. This was to understand which gait class was specifically challenging to identify when presented with other gait impairments. We only used XGBoost for the per-gait classification evaluation, which showed the best performance in the overall classification performance. Our evaluations were done for frontal view-only, sagittal view-only, and frontal and sagittal combined view models. The overall cross-validation scheme was illustrated in Fig 2 (5. LOSO Cross-validation, 5.1 TSFRESH Feature Selection, and 5.3 Model Training).

#### Feature importance analysis.

We applied permutation feature importance analysis to analyze the relevance of each feature for classifying the seven gait classes (NOR, CIR, TRE, ANT, CRO, PAR, and VAU). Permutation importance assessed the impact of each feature on the model’s performance by randomly shuffling the feature values and observing the change in the model’s performance [43]. A positive value indicates the model performance dropped and the feature is therefore important to the model. A negative value indicates the model performance increased and the feature maybe confusing the model. A zero value indicates the model performance is unaffected by the feature.

Concerning the statistical significance of feature importance, we also reported a 95% confidence interval for each feature importance score. We further analyzed the keypoint importance by summing up the feature importance score of all features belonging to each keypoint. This was to understand the overall importance of a particular joint movement for distinguishing different gait classes, agnostic to feature classes.

## Results

### Gait impairment classification

The results from our gait classification experiments are summarized below. Table 3 presents the overall model performance, while Table 4 details the per-class performance of the XGBoost classifier.

**Table 3:** Classification results for multi-class gait impairments. **Bold** text means the best performance in each column. No overlap of confidence intervals shows the difference between values to be statistically significant (p≤0.05)

Model	View	Accuracy	F1 score	Precision	Recall
**SVM**	Frontal	0.376±0.016	0.352±0.016	0.397±0.010	0.376±0.007
	Sagittal	0.640±0.016	0.633±0.015	0.649±0.012	0.640±0.011
	Combined	0.602±0.016	0.591±0.015	0.607±0.013	0.602±0.011
**RF**	Frontal	0.606±0.015	0.601±0.016	0.602±0.015	0.606±0.015
	Sagittal	0.676±0.015	0.672±0.015	0.674±0.012	0.676±0.012
	Combined	0.745±0.014	0.740±0.014	0.742±0.012	0.745±0.012
**XGBoost**	Frontal	0.714±0.015	0.710±0.014	0.713±0.012	0.714±0.011
	Sagittal	0.794±0.013	0.794±0.014	0.796±0.012	0.794±0.012
	Combined	** 0.865±0.011 **	** 0.864±0.013 **	** 0.864±0.012 **	** 0.865±0.011 **

**Table 4:** Classification performance for each gait type using the XGBoost model. **Bold** text indicates the best performance in each column. No overlap of confidence intervals shows the difference between values to be statistically significant (p≤0.05).

Class	Frontal Model			Sagittal Model			Combined Model		
	Precision	Recall	F1-score	Precision	Recall	F1-score	Precision	Recall	F1-score
antalgic	0.630 ± 0.041	0.654 ± 0.042	0.642 ± 0.034	0.646 ± 0.044	0.596 ± 0.043	0.620 ± 0.036	0.857 ± 0.032	0.808 ± 0.033	0.832 ± 0.027
circumduction	**0.926 ± 0.022**	**0.962 ± 0.017**	**0.943 ± 0.016**	0.761 ± 0.041	0.673 ± 0.041	0.714 ± 0.033	**0.927 ± 0.024**	**0.981 ± 0.015**	**0.953 ± 0.015**
crouch	0.627 ± 0.037	0.808 ± 0.031	0.706 ± 0.030	**0.937 ± 0.025**	0.865 ± 0.029	0.900 ± 0.021	0.836 ± 0.031	0.885 ± 0.027	0.860 ± 0.024
normal	0.736 ± 0.039	0.750 ± 0.039	0.743 ± 0.031	0.839 ± 0.032	0.904 ± 0.029	0.870 ± 0.025	0.925 ± 0.026	0.942 ± 0.023	0.933 ± 0.017
Parkinsonian	0.894 ± 0.032	0.808 ± 0.033	0.848 ± 0.026	0.906 ± 0.028	**0.923 ± 0.026**	**0.914 ± 0.019**	0.875 ± 0.028	0.942 ± 0.020	0.907 ± 0.020
Trendelenburg	0.640 ± 0.043	0.615 ± 0.042	0.627 ± 0.035	0.639 ± 0.039	0.750 ± 0.039	0.690 ± 0.031	0.808 ± 0.033	0.808 ± 0.033	0.808 ± 0.028
vaulting	0.538 ± 0.049	0.404 ± 0.041	0.462 ± 0.041	0.846 ± 0.033	0.846 ± 0.033	0.846 ± 0.025	0.818 ± 0.039	0.692 ± 0.041	0.750 ± 0.031

#### Model performance with only frontal view videos as model input.

#### Overall performance.

When given only frontal view input data, the best performing model was XGBoost. Conversely, the SVM model showed the lowest performance.

#### Per-class performance.

The XGBoost model with frontal video views only as inputs showed the highest F1 score for circumduction, F1 score ≥ 0.7 for CIR, CRO, PAR and NOR gaits, F1 score ≥ 0.6 for ANT and TRE gaits, and lowest F1 score for VAU gait.

#### Model performance with only sagittal view videos as input.

#### Overall performance.

XGBoost achieved the highest overall performance when only sagittal view data was given. Similar to frontal view, SVM showed the lowest performance.

#### Per-class performance.

XGBoost showed an F1 score of ≥ 0.90 for CRO and PAR gaits, an F1 score of ≥ 0.85 for NOR and VAU gaits, and lowest F1 score for ANT and TRE gaits.

#### Model performance with combined video view (both frontal and sagittal views) as input.

#### Overall performance.

XGBoost was the best performing model when combined frontal and sagittal views were used for input data. SVM demonstrated the lowest performance. Interestingly, the combined view model in SVM was the only one that performed worse than its single view sagittal counterpart.

#### Per-class performance.

XGBoost showed an F1 score of ≥ 0.9 for CIR, NOR, and PAR gaits, an F1 score of ≥ 0.8 for ANT, CRO, and TRE gaits, and the lowest F1 score for VAU gait. For XGBoost, the per-class performance in combined view models showed either improved or statistically similar performance (within confidence intervals) for six out of seven gait classes compared to the best single view model. The only class that performed worse in the combined view model was VAU, with a decrease in F1-score compared to the sagittal view.

### Model interpretation: Feature and keypoint importance analysis

#### Feature importance.

**Fig 3:**
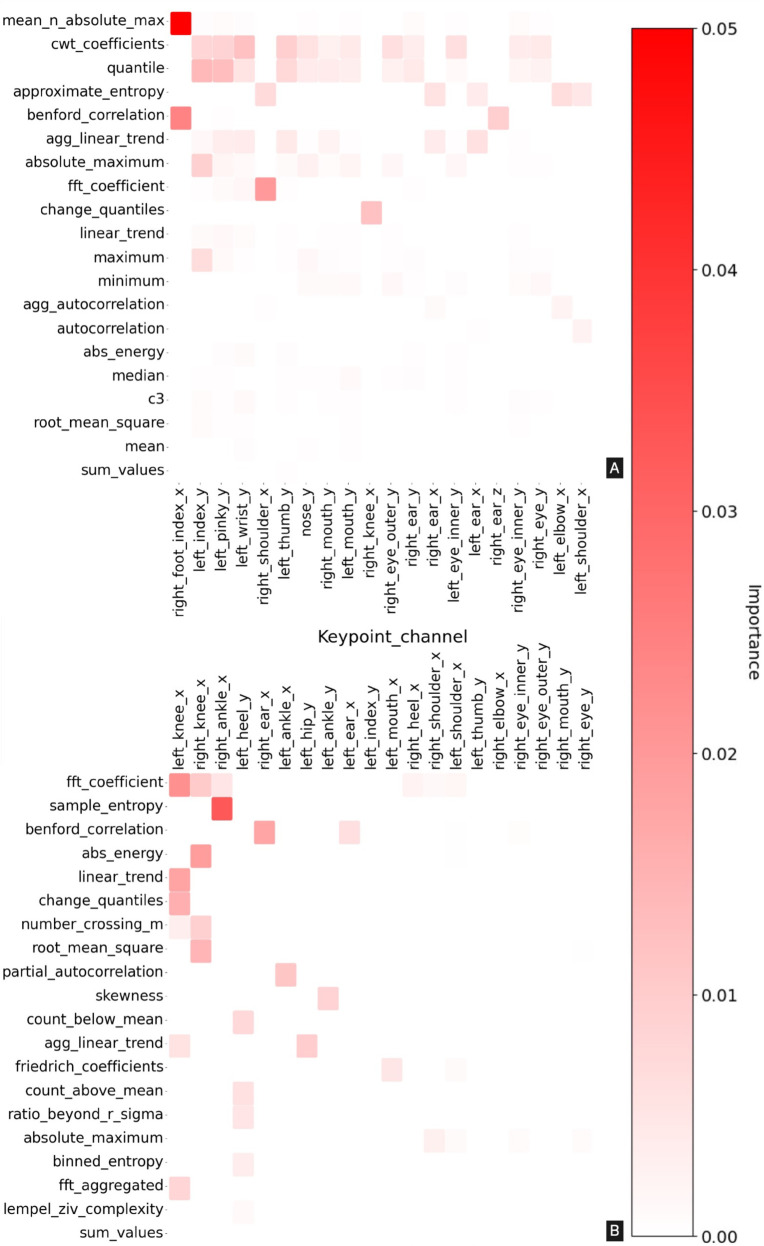
Keypoint feature importance analysis for (A) frontal and (B) sagittal view when classifying seven gait types using XGBoost. The x-axis shows the keypoint and channel (x, y, or z) and the y-axis shows the feature type. Darker color implies higher feature importance in the classification.

In Fig 3, the feature importance analysis for (a) frontal and (b) sagittal view is shown when using XGBoost for multi-class gait classification. The heatmap represents the overall importance of features across keypoint_channels (x-axis) and feature types (y-axis). The heatmap represents the top 20 keypoint_channels and top 20 feature types, ranked by cumulative permutation importance in XGBoost’s performance. The color gradient from white to red in the heatmap indicates the level of importance, with red representing higher significance, while white indicates insignificance to the model classification. Notably, in both heatmaps, most features and keypoint combinations show zero importance.

#### Frontal view videos.

The most relevant features when analyzing frontal view videos were ‘mean_n_absolute_max’ in the x-axis of the right_foot_index_toe; and the ‘cwt_coefficients’ and ‘quantile’ across several keypoints belonging to the hand (fingers), and face (eyes and nose). See Table 1 for definitions of frontal view feature types.

#### Sagittal view videos.

Among the features, ‘fft_coefficient’ for the right_ankle, left_knee and right_knee in the x-axis, which represents the anterio-posterior axis, and ‘sample_entropy’ for the right_ankle in the x-axis stand out as particularly influential, demonstrating high feature importance values. The x-axis of the left_knee and right_knee shows importance across numerous feature types, including: ‘linear_trend’, ‘change_quantiles’, ‘number_crossing_m’ and the earlier mentioned ‘fft_coefficient’. For definitions and the full list of sagittal view feature types see Table 1.

#### Keypoint importance.

**Fig 4:**
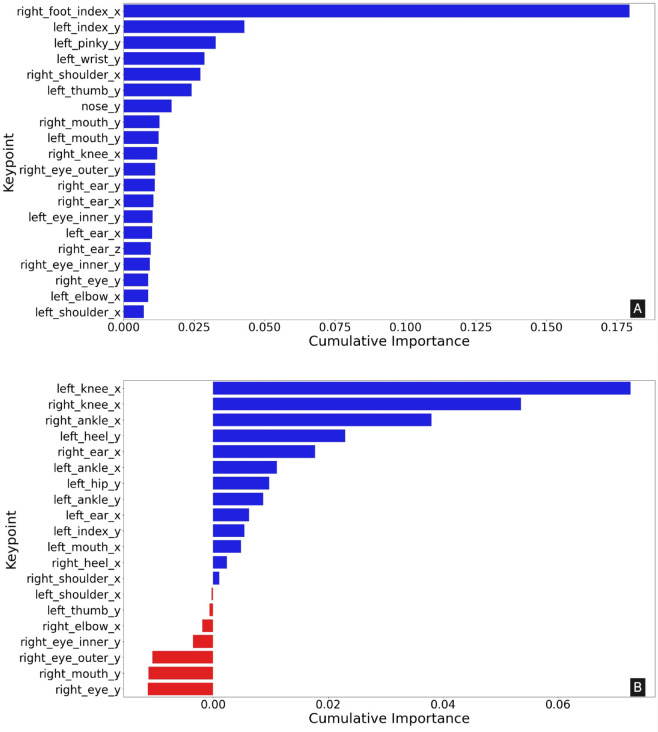
Anatomical keypoint importance analysis in (A) frontal view and (B) sagittal view for classifying seven gait types using XGBoost.

Fig 4 shows the top 20 keypoint importance, based on permutation analysis, aggregating all feature importance values for the corresponding keypoints when using (a) frontal view and (b) sagittal view.

#### Frontal view videos.

Along the frontal view, we find that the x-axis of the right_foot_index is by far the most important, with more than 3× the importance of the next most important keypoint, which is the frontal view y-axis of the left_index. We also notice a greater number of upper limb/body features being important. With the y-axis of left_index, left_pinky, left_wrist, and the x-axis of the right_shoulder all being in the top five of feature importance and scoring more than 0.025.

#### Sagittal view videos.

We find that only the top thirteen keypoints are important to the model from Fig 1(b). Lower limb keypoints like the x-axis of the left_knee, right_knee, right_ankle, and the y-axis of the left_heel are the top four in keypoint importance. The remaining seven keypoints in the top 20 are of negative importance and include only upper body and facial keypoints.

## Discussion

The study highlights the feasibility and effectiveness of a mobile phone-based AI system for classifying multi-class gait impairments, with XGBoost demonstrating superior performance across different video views, particularly when combining frontal and sagittal view video data. Consistent with our hypothesis, we showed that model accuracy improves when we fuse two independently recorded views, even if they are captured at different times. The system’s accuracy benefits from features that capture the frequency, complexity, and directional trends of movements, aligning with clinical observations. These findings underscore the potential of simpler asynchronous multi-view video analysis for improving gait classification and suggest practical applications for privacy-preserving remote monitoring and early detection of gait impairments in clinical settings.

### Gait impairment classification

#### Machine learning models.

The results from Table 3 demonstrate that machine learning model performance varied significantly across different gait video views (frontal, sagittal, and combined frontal + sagittal) and machine learning model types.

XGBoost’s consistent outperformance of both SVM and RF likely stems from its ability to learn nonlinear decision boundaries through iteratively training ensemble classifiers to correct errors made by one classifier to another, making XGBoost robust at handling missing data and outliers [29]. Capturing discriminative patterns across seven gait classes is likely not linearly separable and requires learning non-linear decision boundaries, which is underscored by the poor convergence of linear-kernel SVMs during our experiments.

We applied the Radial Basis Function (RBF) kernel to the SVM after the failure of linear separation, but this kernel was insufficient to project the data into a linearly separable space. As a result, SVM exhibited significantly lower performance compared to XGBoost and RF [44]. Notably, while RF also uses ensemble classifiers, it lacks XGBoost’s iterative process that compensates for errors across classifiers, resulting in lower overall accuracy, a result consistent with prior research showing XGBoost generally outperforming RF in many classification tasks [44].

#### Frontal vs Sagittal vs Combined gait video data as model inputs.

Model performance varied significantly depending on the camera view used (frontal, sagittal, or combined). Models trained on sagittal videos outperformed frontal view models, agreeing with clinical practice, where the sagittal view of a patient’s gait provides greater information to a clinician when diagnosing gait impairments, especially for gait pathologies such as ANT, PAR, and CRO gait. Two gait classes (CIR and ANT) deviated from this trend, performing marginally better in frontal view, likely because their hallmark deviations are more readily visible from the frontal view.

The accuracy of markerless pose estimation relies heavily on detecting and tracking keypoints on the human body [13,14]. These keypoint landmarks serve as reference points that help the algorithm infer the positions of other body parts through skeletal modeling [45]. The mobile phone-based pose estimation model [13] is a lightweight alternative to cloud-server models [14,46], designed to fit the limited computing resources of a phone. Blazepose had a higher error rate in measuring keypoint locations and depth from the camera (z-axis values) compared to the models requiring heavy GPU resources available in cloud servers. We suspect that the lower performance of frontal view video models is due to the inaccuracy of depth information obtained from monocular cameras, which is crucial for evaluating gait in a frontal view. Supporting this, previous studies have shown that sagittal plane kinematics derived from pose estimation algorithms exhibit lower average errors compared to frontal plane counterparts in post-stroke and PAR gaits [12,15].

Inaccuracies in keypoint depth estimation may also contribute to the per-class performance differences observed between frontal and sagittal views. Most notably, detecting CIR requires precise measurement of the semi-circular movement of the lower limb, which is easily captured along the medio-lateral axis and difficult to capture in the sagittal view due to the distinguishing direction of the movement being perpendicular to the camera plane. In line with this, the per-class performance of CIR in the frontal view had a 0.229 higher per-class F1-score compared to the sagittal view model. Similarly, CRO gait is better analyzed by understanding the posture in the sagittal plane, which is poorly captured in the frontal view. This explains the superior performance of XGBoost’s sagittal view in comparison to the frontal view when detecting CRO. As discussed above, the reliability of captured keypoint position data varies between the frontal and sagittal views. By using majority voting on predictions from both the frontal and sagittal view models, the limitations of each view are compensated, leading to improved overall predictions. However, the combined view can be less effective for poorly performing models, such as the frontal view SVM, which has a < 0.40 F1-score, leading to significantly decreased overall prediction quality. This can also be seen in the poor combined view per-class performance of VAU within the XGBoost model, likely caused by the low per-class VAU F1-score of the frontal view model.

### Feature type and keypoint channel importance analysis

#### Features in frontal vs Sagittal views.

The notable features that demonstrated high significance in feature importance analysis were few, as shown in Fig 3. ‘mean_n_absolute_max’ measures the maximum deviation of the right foot from the body center, along the frontal view horizontal axis, during walking. This deviation is an important measure for the gaits captured from the frontal view, like CIR, which are characterized by abnormal deviation of the lower leg away from the body midline in the frontal plane. ‘cwt coefficients’ measure the frequency characteristics of a channel, enabling the identification of subtle changes or periodicities in gait cycle data that distinguish different gait classes. ‘quantiles’ is a statistical measure used to infer the nature of the distribution of points in any time series. ‘approximate_entropy’ quantifies the regularity and complexity of the gait cycle, enabling classifiers to differentiate between steady, predictable walking and irregular behaviors that may discriminate between specific gait impairments. ‘agg_linear_trend’ captures the overall directional movement of keypoint time series, such as forward hip progression or vertical knee oscillation, helping to identify consistent gait characteristics crucial for classification. Finally, ‘absolute_maximum’ measures the peak values of keypoint time series during the gait cycle, which are critical for distinguishing different walking speeds or intensities.

Along the sagittal plane video data, the features like ‘fft_coefficient’ and ‘sample_entropy’ were particularly influential in the x axes of the left knee, right knee, and right ankle. The importance of the ‘sample_entropy’ of the right ankle’s x-axis was the highest and ≥ 0.03. The ‘fft_coefficient’ and ‘sample_entropy’ provide detailed information on the frequency and regularity of movements, which may be vital for understanding motor control, and inform future research and the design of data-driven clinical decision-making algorithms for gait diagnosis. The standout features–such as ‘fft_coefficients’, ‘sample_entropy’, and ‘linear_trends’– demonstrate the importance of capturing both the frequency and complexity of movements across specific body parts. For example, ‘fft_coefficients’ are highly relevant for joints like the ankle and knee, indicating that repetitive or cyclic patterns in lower body motion play a significant role in the model’s predictions. These movements could correspond to gait frequency, or other repeated actions, all of which are important in gait analysis [47,48]. The entropy-based features, such as ‘sample_entropy’ and ‘binned_entropy’, highlight the need to capture the unpredictability and complexity in motion, suggesting that irregular or erratic movements may be key indicators of certain classes [49,50]. Overall, the model performances appear to leverage feature types that capture the frequency, complexity, and directional trends of movement, most of which have been shown to be meaningful in gait analysis and align with clinical intuition. Our results show that a model trained on features extracted without gait expert input also focuses on and prioritizes aspects of gait characteristics considered important in clinical practice.

#### Keypoints in Frontal vs Sagittal views.

Other than the right foot index, the frontal view had a very high permutation importance in the y-axis of the left index finger, the left pinky, and the left wrist, and the x-axis of the right shoulder.

As mentioned above, the high importance of the right foot keypoint can be attributed to its relevance in classifying gaits like CIR, where quantifying deviation of the lower limb from the midline of the body is very helpful [51,52]. Furthermore, upperlimb movement has also been shown to be important for balance, posture, and gait coordination in NOR gait [53]. The high importance of the contralateral y-axis upperlimb keypoints indicate their utility in capturing compensatory movements and imbalance in the gaits. The keypoint importance analysis revealed that the top five features in the sagittal view included lower limb keypoints, in order of importance, they are the x-axis of the left knee, right knee, and right ankle, followed by the y-axis of the left heel. It also includes the right ear’s x-axis with an importance > 0.02. The importance of keypoints in the lower body (e.g., ankles and knees) along with facial keypoints (as is the ear) suggests that the model is sensitive to not only lowerlimb positions but also the relative posture of the head. Lower limb movements often reflect changes in balance, gait, and physical effort, while keypoints of the head, along with the similarly important hip, are indicative of postural height or the height as measured during the subjects gait, which is closely tied to gait classes like CRO and PAR.

### Limitations and future direction

This study faces several limitations that will be addressed in our future research. First, our dataset was simulated by able-bodied participants with clinical training, rather than by individuals with real gait pathologies. Although this approach allowed controlled capture of seven reasonably realistic and distinct gait patterns, it cannot fully replicate the variability, compensatory motions, and severity found in real patient populations. We consider the current study as an important step to develop gait classification models using high quality curated gait video datasets. Our ongoing work is recruiting patients with real gait impairments from various demographic groups and clinical diagnoses to further validate the generalizability of our proposed framework. This will help quantify the clinical utility of a pretrained model with a simulated gait dataset.

Second, to maintain standardization of methodology, all asymmetrical gait impairments in this work were mimicked as right-leg deficits (e.g., circumduction, antalgic gait on the right side). Consequently, our feature-importance analysis, highlighting ‘mean_n_absolute_max’ of the right-foot index in the x-axis, may partly reflect this design choice. This bias introduces a potential limitation of the trained model for individuals with left-sided impairments. This is concerning as hemiplegic (or unilateral) gaits consist of a large portion of certain impaired gait patterns, such as those following stroke [54] with significant heterogeneity affecting either the right or left side of the gaits [55]. Future work will collect larger sample sizes, simulating unilateral impairments on either the right or left side for analysis and test the model’s generalizability.

Third, the relatively small number of participants and gait classes limits generalizability. The current classification system assumes mutually exclusive categories, whereas in reality, gait impairments often arise from overlapping conditions, with the same individual showing multiple gait classes or patterns, leading to more complex presentations that are not fully reflected in our model. For example, in real-world clinical settings, a video of the same individual may demonstrate three different gait classes (e.g., CIR, VAU, and ANT gait) with varying levels of severity. Expanding the dataset to include multi-label examples and a broader demographic will help models handle complex, real-world presentations. Future work can also employ generative AI methods to produce synthetic mixed condition gait, allowing the model to train and generalize better on this subgroup of the population.

Fourth, we employed classical machine learning classifiers (XGBoost, RF, SVM) on hand-engineered time-series features. While interpretable and efficient, these “shallow” methods lack the representational power of modern deep architectures such as DeepConvLSTMs [56], attention-based networks [57], Graph convolutional Networks, [58] or Transformer models [59]. These advanced models can be trained on much larger datasets and, as a result, may also offer superior explainability of a wide variety of conditions in a generalized manner.

Explainable techniques like attention-based approaches and Shapley values [60], can provide deeper insights into the decision-making process behind their gait classification.

Fifth, our time-series features aggregate over the full trial without considering the number of gait cycles needed for quantifying each gait type. Our current analyses do not isolate the effect of cycle count on model performance; instead, the classifiers are trained on sliding one second windows with 50% overlap, which contain fewer than two complete cycles on average. A comprehensive investigation of how window length or cycle count influences model performance would be a valuable direction for future work.

Finally, all recordings were captured under controlled instructions and camera setups. To move toward clinical deployment, models must be tested on truly “in-the-wild” videos with realistic clinical and at-home settings captured in various backgrounds, clothing, patient demographics, and camera positions. Incorporating diverse real-world data will be critical for robust, privacy-preserving gait monitoring in clinical, home, and community settings.

### Clinical applications

The proposed mobile phone-based, privacy-preserving system has strong potential for clinical applications in gait analysis, particularly for early and more accurate diagnosis. The on-device processing ensures privacy, making the system suitable for use in non-clinical environments like outdoor community settings or at home without the need for costly or complex instrumentation, and in the context of tele-rehabilitation.

The proposed system’s scalability, privacy-preservation, and computational simplicity make it an ideal solution for remotely monitoring high-risk populations, such as elderly individuals in nursing homes who are prone to gait impairments. By enabling continuous assessment without requiring frequent clinic visits, the system can monitor patient’s gait over time periods much longer than a ordinary clinician would be exposed to. Thus, these methods can enable clinician-researcher teams to detect gait impairments early and to flag them for timely treatment before serious mobility loss. Early identification of subtle pre-clinical gait impairments will allow clinical teams to prioritize and allocate therapist resources more efficiently, ensuring that patients who need rehabilitative care are identified and treated sooner.

Active collaboration between clinicians, machine learning engineers, and software developers is critical to ensure the usability and convenience of such tools. Both clinician-friendly and patient-friendly interfaces are needed to further facilitate the adoption of this technology in routine practice. Ultimately, this technology has the potential to enhance the accuracy and accessibility of gait analysis, improving patient outcomes in both clinical and home-based rehabilitation settings.

### Conclusions

This study demonstrates the effectiveness of a mobile phone-based, privacy-preserving AI system for classifying simulated gait impairments. Using pose estimation models and multi-view videos, XGBoost with combined frontal and sagittal views offers the highest accuracy (0.864 F1 score). Sagittal views were generally more effective, but certain gait classes like CIR benefited from frontal views, emphasizing the value of a multi-view approach with asynchronous video. Key features such as FFT coefficients and entropy-based measures proved critical for distinguishing between gait classes by capturing the frequency and complexity of movements. The on-device pose estimation ensures privacy, making this system scalable for real-world applications, such as home-based rehabilitation. Future work should validate these findings on real-world, diverse clinical datasets and explore advanced models for further improvements. Overall, this system offers a practical solution for accessible and interpretable gait analysis, supporting early detection and personalized rehabilitation.

## References

[1] Wennberg AMV, Savica R, Mielke MM. Association between various brain pathologies and gait disturbance. Dement Geriatr Cogn Disord. 2017;43(3–4):128–43. doi: 10.1159/000456541 28152532 PMC5466166

[2] Harris GF, Wertsch JJ. Procedures for gait analysis. Archives of Physical Medicine and Rehabilitation. 1994;75(2):216–25. doi: 10.1016/0003-9993(94)90399-9 8311681

[3] Coutts F. Gait analysis in the therapeutic environment. Man Ther. 1999;4(1):2–10. doi: 10.1016/s1356-689x(99)80003-4 10463015

[4] Perry J, JMB. Gait analysis: normal and pathological function. Journal of Sports Science & Medicine. 2010;9(2):353.

[5] Krebs DE, Edelstein JE, Fishman S. Reliability of observational kinematic gait analysis. Phys Ther. 1985;65(7):1027–33. doi: 10.1093/ptj/65.7.1027 3892553

[6] Brunnekreef JJ, van Uden CJT, van Moorsel S, Kooloos JGM. Reliability of videotaped observational gait analysis in patients with orthopedic impairments. BMC Musculoskelet Disord. 2005;6:17. doi: 10.1186/1471-2474-6-17 15774012 PMC555760

[7] Kay RM, Dennis S, Rethlefsen S, Reynolds RA, Skaggs DL, Tolo VT. The effect of preoperative gait analysis on orthopaedic decision making. Clin Orthop Relat Res. 2000;(372):217–22. doi: 10.1097/00003086-200003000-00023 10738430

[8] McGrath M, Wood J, Walsh J, Window P. The impact of three-dimensional gait analysis in adults with pathological gait on management recommendations. Gait Posture. 2023;105:75–80. doi: 10.1016/j.gaitpost.2023.06.014 37490826

[9] Mukaino M, Ohtsuka K, Tanikawa H, Matsuda F, Yamada J, Itoh N, et al. Clinical-oriented three-dimensional gait analysis method for evaluating gait disorder. J Vis Exp. 2018;(133):57063. doi: 10.3791/57063 29553535 PMC5931438

[10] Hulleck AA, Menoth Mohan D, Abdallah N, El Rich M, Khalaf K. Present and future of gait assessment in clinical practice: towards the application of novel trends and technologies. Front Med Technol. 2022;4:901331. doi: 10.3389/fmedt.2022.901331 36590154 PMC9800936

[11] Sato K, Nagashima Y, Mano T, Iwata A, Toda T. Quantifying normal and parkinsonian gait features from home movies: Practical application of a deep learning-based 2D pose estimator. PLoS One. 2019;14(11):e0223549. doi: 10.1371/journal.pone.0223549 31725754 PMC6855634

[12] Stenum J, Rossi C, Roemmich RT. Two-dimensional video-based analysis of human gait using pose estimation. PLoS Comput Biol. 2021;17(4):e1008935. doi: 10.1371/journal.pcbi.1008935 33891585 PMC8099131

[13] Bazarevsky V, Grishchenko I, Raveendran K, Zhu T, Zhang F, Grundmann M. BlazePose: on-device real-time body pose tracking. arXiv preprint 2020. http://arxiv.org/abs/2006.10204

[14] Cao Z, Hidalgo G, Simon T, Wei SE, Sheikh Y. OpenPose: realtime multi-person 2D pose estimation using part affinity fields. arXiv preprint 2019. http://arxiv.org/abs/1812.08008 10.1109/TPAMI.2019.2929257 31331883

[15] Stenum J, Hsu MM, Pantelyat AY, Roemmich RT. Clinical gait analysis using video-based pose estimation: multiple perspectives, clinical populations, and measuring change. PLOS Digit Health. 2024;3(3):e0000467. doi: 10.1371/journal.pdig.0000467 38530801 PMC10965062

[16] Cimorelli A, Patel A, Karakostas T, Cotton RJ. Validation of portable in-clinic video-based gait analysis for prosthesis users. Sci Rep. 2024;14(1):3840. doi: 10.1038/s41598-024-53217-7 38360820 PMC10869722

[17] Kidziński Ł, Yang B, Hicks JL, Rajagopal A, Delp SL, Schwartz MH. Deep neural networks enable quantitative movement analysis using single-camera videos. Nat Commun. 2020;11(1):4054. doi: 10.1038/s41467-020-17807-z 32792511 PMC7426855

[18] Gholami M, Ward R, Mahal R, Mirian M, Yen K, Park KW, et al. Automatic labeling of Parkinson’s disease gait videos with weak supervision. Med Image Anal. 2023;89:102871. doi: 10.1016/j.media.2023.102871 37480795

[19] Cost of a Data Breach Report 2024 . 2024. https://www.ibm.com/reports/data-breach

[20] Lugaresi C, Tang J, Nash H, McClanahan C, Uboweja E, Hays M. MediaPipe: a framework for building perception pipelines. arXiv preprint 2019. http://arxiv.org/abs/1906.08172

[21] Gandbhir VN, Lam JC, Lui F, Rayi A. Trendelenburg Gait. StatPearls. Treasure Island (FL): StatPearls Publishing; 2025. 31082138

[22] Awad LN, Bae J, Kudzia P, Long A, Hendron K, Holt KG, et al. Reducing circumduction and hip hiking during hemiparetic walking through targeted assistance of the paretic limb using a soft robotic exosuit. Am J Phys Med Rehabil. 2017;96(10 Suppl 1):S157–64. doi: 10.1097/PHM.0000000000000800 28777105 PMC7479995

[23] Hausdorff JM. Gait dynamics in Parkinson’s disease: common and distinct behavior among stride length, gait variability, and fractal-like scaling. Chaos. 2009;19(2):026113. doi: 10.1063/1.3147408 19566273 PMC2719464

[24] Villa C, Drevelle X, Bonnet X, Lavaste F, Loiret I, Fodé P, et al. Evolution of vaulting strategy during locomotion of individuals with transfemoral amputation on slopes and cross-slopes compared to level walking. Clin Biomech (Bristol). 2015;30(6):623–8. doi: 10.1016/j.clinbiomech.2015.03.022 25843483

[25] Pandey RA, Johari AN, Shetty T. Crouch gait in cerebral palsy: current concepts review. Indian J Orthop. 2023;57(12):1913–26. doi: 10.1007/s43465-023-01002-5 38009172 PMC10673808

[26] Bulling A, Blanke U, Schiele B. A tutorial on human activity recognition using body-worn inertial sensors. ACM Comput Surv. 2014;46(3):1–33. doi: 10.1145/2499621

[27] Crammer K, Singer Y. On the algorithmic implementation of multiclass kernel-based vector machines. Journal of Machine Learning Research. 2001;2(Dec):265–92.

[28] Breiman L. Random forests. Machine Learning. 2001;45(1):5–32. doi: 10.1023/a:1010933404324

[29] Chen T, Guestrin C. XGBoost. In: Proceedings of the 22nd ACM SIGKDD International Conference on Knowledge Discovery and Data Mining. 2016. p. 785–94. 10.1145/2939672.2939785

[30] Hammerla NY, Kirkham R, Andras P, Ploetz T. On preserving statistical characteristics of accelerometry data using their empirical cumulative distribution. In: Proceedings of the 2013 International Symposium on Wearable Computers. 2013. p. 65–8. 10.1145/2493988.2494353

[31] Christ M, Braun N, Neuffer J, Kempa-Liehr AW. Time series FeatuRe extraction on basis of scalable hypothesis tests (tsfresh – a python package). Neurocomputing. 2018;307:72–7. doi: 10.1016/j.neucom.2018.03.067

[32] Christ M, Kempa-Liehr AW, Feindt M. Distributed and parallel time series feature extraction for industrial big data applications. arXiv preprint 2017. http://arxiv.org/abs/1610.07717

[33] Benjamini Y, Yekutieli D. The control of the false discovery rate in multiple testing under dependency. Ann Statist. 2001;29(4). doi: 10.1214/aos/1013699998

[34] Chawla NV, Japkowicz N, Kotcz A. Editorial. SIGKDD Explor Newsl. 2004;6(1):1–6. doi: 10.1145/1007730.1007733

[35] Chawla NV, Bowyer KW, Hall LO, Kegelmeyer WP. SMOTE: synthetic minority over-sampling technique. jair. 2002;16:321–57. doi: 10.1613/jair.953

[36] Noh B, Youm C, Goh E, Lee M, Park H, Jeon H, et al. XGBoost based machine learning approach to predict the risk of fall in older adults using gait outcomes. Sci Rep. 2021;11(1):12183. doi: 10.1038/s41598-021-91797-w 34108595 PMC8190134

[37] Gong NJ, Clifford GD, Esper CD, Factor SA, McKay JL, Kwon H. Classifying tremor dominant and postural instability and gait difficulty subtypes of Parkinson’s disease from full-body kinematics. Sensors (Basel). 2023;23(19):8330. doi: 10.3390/s23198330 37837160 PMC10575216

[38] Kim J-K, Bae M-N, Lee K, Kim J-C, Hong SG. Explainable artificial intelligence and wearable sensor-based gait analysis to identify patients with osteopenia and sarcopenia in daily life. Biosensors (Basel). 2022;12(3):167. doi: 10.3390/bios12030167 35323437 PMC8946270

[39] Wainer J, Cawley G. Nested cross-validation when selecting classifiers is overzealous for most practical applications. Expert Systems with Applications. 2021;182:115222. doi: 10.1016/j.eswa.2021.115222

[40] Parvandeh S, Yeh H-W, Paulus MP, McKinney BA. Consensus features nested cross-validation. Bioinformatics. 2020;36(10):3093–8. doi: 10.1093/bioinformatics/btaa046 31985777 PMC7776094

[41] Akiba T, Sano S, Yanase T, Ohta T, Koyama M. Optuna. In: Proceedings of the 25th ACM SIGKDD International Conference on Knowledge Discovery & Data Mining. 2019. p. 2623–31. 10.1145/3292500.3330701

[42] Binomial proportion confidence interval. 2024. https://en.wikipedia.org/w/index.php?title=Binomial_proportion_confidence_interval&oldid=1223672356

[43] Altmann A, Toloşi L, Sander O, Lengauer T. Permutation importance: a corrected feature importance measure. Bioinformatics. 2010;26(10):1340–7. doi: 10.1093/bioinformatics/btq134 20385727

[44] Bentéjac C, Csörgő A, Martínez-Muñoz G. A comparative analysis of gradient boosting algorithms. Artif Intell Rev. 2020;54(3):1937–67. doi: 10.1007/s10462-020-09896-5

[45] Felzenszwalb PF, Huttenlocher DP. Pictorial structures for object recognition. International Journal of Computer Vision. 2005;61(1):55–79. doi: 10.1023/b:visi.0000042934.15159.49

[46] Toshev A, Szegedy C. DeepPose: human pose estimation via deep neural networks. In: 2014 IEEE Conference on Computer Vision and Pattern Recognition. 2014. p. 1653–60. 10.1109/cvpr.2014.214

[47] Winner TS, Rosenberg MC, Jain K, Kesar TM, Ting LH, Berman GJ. Discovering individual-specific gait signatures from data-driven models of neuromechanical dynamics. PLoS Comput Biol. 2023;19(10):e1011556. doi: 10.1371/journal.pcbi.1011556 37889927 PMC10610102

[48] Winner TS, Rosenberg MC, Berman GJ, Kesar TM, Ting LH. Gait signature changes with walking speed are similar among able-bodied young adults despite persistent individual-specific differences; 2024. https://www.biorxiv.org/content/10.1101/2024.05.01.591976v1 10.1038/s41598-024-70787-8 PMC11345452 39183361

[49] Lockhart T, Stergiou N. New perspectives in human movement variability. Ann Biomed Eng. 2013;41(8):1593–4. doi: 10.1007/s10439-013-0852-0 23797778 PMC3758411

[50] Stergiou N, Decker LM. Human movement variability, nonlinear dynamics, and pathology: is there a connection?. Hum Mov Sci. 2011;30(5):869–88. doi: 10.1016/j.humov.2011.06.002 21802756 PMC3183280

[51] Stanhope VA, Knarr BA, Reisman DS, Higginson JS. Frontal plane compensatory strategies associated with self-selected walking speed in individuals post-stroke. Clin Biomech (Bristol). 2014;29(5):518–22. doi: 10.1016/j.clinbiomech.2014.03.013 24768223 PMC4367535

[52] Tyrell CM, Roos MA, Rudolph KS, Reisman DS. Influence of systematic increases in treadmill walking speed on gait kinematics after stroke. Phys Ther. 2011;91(3):392–403. doi: 10.2522/ptj.20090425 21252308 PMC3048817

[53] Meyns P, Bruijn SM, Duysens J. The how and why of arm swing during human walking. Gait Posture. 2013;38(4):555–62. doi: 10.1016/j.gaitpost.2013.02.006 23489950

[54] Li S, Francisco GE, Zhou P. Post-stroke hemiplegic gait: new perspective and insights. Front Physiol. 2018;9:1021. doi: 10.3389/fphys.2018.01021 30127749 PMC6088193

[55] Hedna VS, Bodhit AN, Ansari S, Falchook AD, Stead L, Heilman KM, et al. Hemispheric differences in ischemic stroke: is left-hemisphere stroke more common?. J Clin Neurol. 2013;9(2):97–102. doi: 10.3988/jcn.2013.9.2.97 23626647 PMC3633197

[56] Ordóñez FJ, Roggen D. Deep convolutional and LSTM recurrent neural networks for multimodal wearable activity recognition. Sensors (Basel). 2016;16(1):115. doi: 10.3390/s16010115 26797612 PMC4732148

[57] Singh SP, Lay-Ekuakille A, Gangwar D, Sharma MK, Gupta S. Deep ConvLSTM with self-attention for human activity decoding using wearables. arXiv preprint 2020. http://arxiv.org/abs/2005.00698

[58] Yan S, Xiong Y, Lin D. Spatial temporal graph convolutional networks for skeleton-based action recognition. arXiv preprint 2018. http://arxiv.org/abs/1801.07455

[59] Xu Y, Zhang J, Zhang Q, Tao D. ViTPose: simple vision transformer baselines for human pose estimation. arXiv preprint 2022. http://arxiv.org/abs/2204.12484

[60] Sundararajan M, Najmi A. The many shapley values for model explanation. In: Proceedings of the 37th International Conference on Machine Learning. 2020. p. 9269–78. https://proceedings.mlr.press/v119/sundararajan20b.html

